# Plasma DNA Mediate Autonomic Dysfunctions and White Matter Injuries in Patients with Parkinson's Disease

**DOI:** 10.1155/2017/7371403

**Published:** 2017-01-23

**Authors:** Meng-Hsiang Chen, Pei-Chin Chen, Cheng-Hsien Lu, Hsiu-Ling Chen, Yi-Ping Chao, Shau-Hsuan Li, Yi-Wen Chen, Wei-Che Lin

**Affiliations:** ^1^Department of Diagnostic Radiology, Kaohsiung Chang Gung Memorial Hospital, Kaohsiung, Taiwan; ^2^Chang Gung University College of Medicine, Kaohsiung, Taiwan; ^3^Department of Neurology, Kaohsiung Chang Gung Memorial Hospital, Kaohsiung, Taiwan; ^4^Graduate Institute of Medical Mechatronics, Chang Gung University, Taoyuan, Taiwan; ^5^Department of Neurology, Chang Gung Memorial Hospital, Linkou, Taoyuan, Taiwan; ^6^Department of Medicine, Kaohsiung Chang Gung Memorial Hospital, Kaohsiung, Taiwan

## Abstract

*Background*. Cardiovascular autonomic dysfunction is well known in Parkinson's disease (PD) presentation and it produces hypoperfusion of vital organs. The association between cardiovascular autonomic dysfunction and oxidative stress was examined in previous animal models. Oxidative stress and neuroinflammation were thought to have roles in PD pathogenesis. Owing to the relative low intrinsic antioxidative properties, brain white matter (WM) is vulnerable to the oxidative stress. This study is conducted to examine possible relationships by using a hypothesis-driven mediation model.* Methods*. Twenty-nine patients with PD and 26 healthy controls participated in this study, with complete examinations of cardiac autonomic parameters, plasma DNA level, and WM integrity. A single-level three-variable mediation model was used to investigate the possible relationships.* Results*. The elevated serum oxidative stress biomarkers include plasma nuclear DNA and mitochondrial DNA, and poorer cardiac autonomic parameters and multiple regional microstructural WM changes are demonstrated. Further mediation analysis shows that plasma nuclear DNA served as the mediators between poorer baroreflex sensitivity and mean diffusivity changes in cingulum.* Conclusions*. These results provide a possible pathophysiology for how the poor baroreflex sensitivity and higher oxidative stress adversely impacted the WM integrity. This model could provide us with a piece of the puzzle of the entire PD pathogenesis.

## 1. Introduction

Cardiovascular autonomic dysfunction is well known in Parkinson's disease (PD) presentation and may be a leading cause of life-threatening processes [1]. Baroreflex sensitivity (BRS) testing using the autoregressive analysis of cyclic changes in R-R intervals to produce power spectra could be a reflection of autonomic influences on heart rate and blood pressure [2]. In previous studies, it has been suggested that poorer BRS status was observed in PD patients [3] and produced hypoperfusion of vital organs [4].

The influences of hypoperfusion may be observed in various diseases, even mild diseases such as obstructive sleep apnea [5]. Cells suffering from a hypoxic environment in hypoperfusion tend to experience apoptosis or cell death due to impairment of the mitochondrial function [6]. This process generates considerable oxidative stress and releases plasma DNA, including mitochondrial DNA and nuclear DNA, to serum. As an end result of oxidative stress, the plasma DNA level in peripheral blood could be representative of the oxidative stress status in these PD patients. This provides us with an opportunity to detect possible CNS changes in peripheral blood.

The association between cardiovascular autonomic dysfunction and oxidative stress was examined in previous animal models [7] and oxidative stress and neuroinflammation were thought to have roles in PD pathogenesis [8]. Owing to relatively low intrinsic antioxidative properties [9], brain white matter (WM) is vulnerable to oxidative stress. Combined with the generation of reactive oxygen species of lipid-rich myelin [10], oxidative stress plays a more significant role in brain WM damage. Recent studies show that not only the neurotoxic potential of microglia [11] but also the repairing character of the oligodendrocytes [12] is all influenced by oxidative stress status. These causes are believed to have dramatic impact on brain WM and present as the deficits of DTI (diffusion tensor imaging) indices.

Diffusion tensor imaging (DTI) is a noninvasive method used to detect the microstructural damage of brain WM [13]. Fractional anisotropy (FA) and mean diffusivity (MD) are the most well-known indices for evaluating the integrity and diffusivity values [14]. Furthermore, the axial diffusivity (AD) and radial diffusivity (RD) derived from FA could help us to determine the different WM pathologies, such as gliosis or myelin destruction [5]. However, there have still been only a limited number of reports indicating which DTI indices best reflect the damage caused by oxidative stress.

Based on previous reports, poor cardiovascular autonomic function could induce hypoperfusion [4] and result in apoptosis or cell death due to impaired mitochondrial function [6]. This process generates considerable oxidative stress and releases plasma DNA, including mitochondrial DNA and nuclear DNA. Additionally, oxidative stress has been linked to poor cardiovascular autonomic function [7] and degenerative brain changes under neuroinflammation [8]. These clues imply that there may be some causal relationship between poor cardiovascular autonomic function, oxidative stress, and WM damage. We propose that (1) compared with controls PD patients show lower cardiovascular autonomic activity, higher plasma DNA levels, and poorer white matter DTI indices; (2) associations among the reduced autonomic function, increased plasma DNA level, and reduced brain WM DTI indices existed; additionally (3) we applied a hypothesis-driven mediation model to explore the possible interactions of autonomic dysfunction, elevated plasma DNA levels, and WM declined in PD.

## 2. Methods

### 2.1. Participants

Protocols of this study were approved by the Local Ethics Committee on Human Research of Kaohsiung Chang Gung Memorial Hospital in Taiwan. The written informed consent from the participants was gathered prior to participation in this study. Twenty-nine patients (20 men and 9 women, mean age: 61.51 ± 8.27 years) with no previous underlying disease of neurology or psychology, neurological or psychotropic medication, or other contraindication for high magnetic fields such as receiving magnetic resonance imaging (MRI) were enrolled at the Neurology Outpatient Department prospectively. All patients were diagnosed by experienced neurologists according to Parkinson's Disease Society's criteria [15], the Unified Parkinson Disease Rating Scale (UPDRS) [16], and modified Hoehn and Yahr staging (H&Y) scale [17], in the “OFF” state. The UPDRS is evaluated via clinical observation and interview for multiple aspects of PD, such as mental dysfunction and mood (Part I), motor disability (Part II), motor impairment (Part III), and treatment-related motor and nonmotor complications (Part IV). The modified H&Y scale (from stages 1 through 5, more severe in later stages) could provide evaluation of functional disability according to clinical findings and is commonly used to track the progression of Parkinson's disease. The Schwab and England (S&E) scale was used to record the subjects' abilities, wherein 100% indicates a completely independent person, while a score of 0% indicates a person with vegetative functions.

26 sex- and age-matched healthy subjects (19 men and 7 women, mean age: 60.11 ± 7.77 years) with similar levels of education but no medical history of brain trauma, substance abuse, neurological diseases, or psychiatric illnesses were also enrolled for comparison.

### 2.2. Cardiovascular Autonomic Parameters

Continuously recorded standard three-lead ECG (Ivy Biomedical, model 3000; Branford, CT) was used for heart rate derivation. Beat-to-beat photoplethysmographic recording (Finameter Pro, Ohmeda; Englewood, OH) is used for recording arterial blood pressure. The heart rate response to deep breathing (HRDB), Valsalva ratio (VR), and baroreflex sensitivity (BRS) were measured using Testworks software (WR Medical Electronics Company, Stillwater, MN).

The R-R interval differences of beat to beat were interpolated using a third-order polynomial, and the resampled interval was 0.5 sec. The Fourier transformation was applied for signal (512 samples) processing to fit the frequency domain. According to the spectral powers, these signals were divided into three frequency domains. 0.15–0.4 Hz was defined as high frequency (HF), 0.04–0.15 Hz was set as low frequency (LF), and 0–0.04 Hz was regarded as very low frequency (VLF).

### 2.3. MR Image Acquisition

#### 2.3.1. Data Acquisition

The 3.0 tesla whole-body GE Signa MRI system (General Electric Healthcare, Milwaukee, WI, USA) with an eight-channel head coil was used for MRI scanning. Along the anterior-posterior commissural line (AC-PC line) in the axial plane, DTI was conducted using a single shot echo-planar imaging (EPI) sequence (repetition time/echo time [TR/TE] = 15,800/77 ms, voxel size = 1 × 1 × 2.5 mm^3^, field of view [FOV] = 25.6 cm, matrix size = 256 × 256, number of excitations [NEX] = 3, and 55 slices without gaping). The gradient encoding schemes are 13 noncollinear directions (*b*-value of 1000 s/mm^2^) and a nondiffusion-weighted image volume (*b*-value of 0 s/mm^2^) in diffusion images.

T1-weighted images are scanned along the AC-PC line by a three-dimensional fluid-attenuated inversion-recovery fast spoiled gradient recalled echo sequence (3D IR-FSPGR, TR/TE/inversion time = 9.5/3.9/450 ms, flip angle = 20°, voxel size = 0.47 × 0.47 × 1.3 mm^3^, FOV = 25.6 cm, matrix size = 512 × 512, and 110 slices without gaping). Axial T2-weighted images were scanned by a fast spin-echo sequence (TR/TE = 4,200/102 ms, FOV = 24 cm, matrix size = 320 × 256, NEX = 2, echo train length = 18, and slice thickness = 5 mm). The T1- and T2-weighted images were used to check for any intracranial abnormality. This protocol required twenty minutes for each participant.

### 2.4. Laboratory Measurement of Inflammatory Markers in the Peripheral Circulation

#### 2.4.1. Blood Sampling

Plasma levels of nuclear and mitochondrial DNA were examined in all subjects as oxidative stress biomarkers. Serum was collected by venipuncture from the forearm on the same day as the cardiovascular autonomic examinations and MRI study.

#### 2.4.2. Determination of Plasma Nuclear DNA and Mitochondrial DNA

Three ml of peripheral venous blood was gathered in ethylenediaminetetraacetic acid-containing tubes. The procedural details were as described in a previous study [18]. The blood samples were initially centrifuged; then the plasma was transferred to polypropylene tubes and centrifuged again to ensure cell-free specimen collection. According to the manufacturer's blood and body fluid protocol, a QIAamp Blood Kit (Qiagen, Hilden, Germany) was used for DNA extraction and calculating the target DNA concentration. A real-time quantitative polymerase chain reaction (RT-PCR) assay (Roche LightCycler, Roche, Grenzach-Wyhlen, Germany) was used for detecting the*β-globin* and* ND2* genes as plasma nuclear and mitochondrial DNA. Continuous measurements of Sybr green fluorescent dye bound to double-stranded DNA generated in quantitative RT-PCR were done as expression of plasma DNA. Human genomic DNA (Roche) facilitates the DNA standard curve and quantitative results are expressed as ng/ml.

### 2.5. Data Analysis

#### 2.5.1. MRI DTI Indices Data Processing

The preprocessing of magnetic resonance images was done using the FSL v5.0.4 (Functional Magnetic Resonance Imaging of the Brain (FMRIB) Software Library, Oxford, UK) [19]. The nondiffusion-weighted image was facilitated for registration of diffusion-weighted image by an affine registration approach in FMRIB's Linear Image Registration Tool (FLIRT; part of FSL) for minimizing image distortion from eddy currents and simple head motion. The non-brain tissue and background noise in images were removed using the Brain Extraction Tool (BET; part of FSL). A diffusion tensor model was used for voxel fitting for the calculation of FA values using FMRIB's Diffusion Toolbox (FDT, part of FSL). These preprocessing methods have also been reported in previous papers [20–24].

The standard template space for group comparisons is set in the Montreal Neurological Institute (MNI) space. The FMRIB58_FA standard space image in FSL was used as the target image. Each FA image was normalized spatially to the target image with FMRIB's Nonlinear Image Registration Tool (FNIRT, part of FSL). Finally, the voxel size of each image was resampled to 1 mm^3^ resolution. An 8 mm Gaussian kernel was used in all normalized FA images for image smoothing.

### 2.6. Statistical Analysis

#### 2.6.1. Analysis of Demographic Data, Cardiovascular Autonomic Parameters, DTI, and Plasma DNA

All demographic data were compared between the groups using the two-sample Student *t*-test and Pearson's chi-square test and are reported as the mean ± the standard deviation (SD). Analysis of covariance (ANCOVA) was used to analyze differences in cardiovascular autonomic parameters and plasma DNA levels (with the participant's age and sex as covariates) and differences in DTI indices (with the participant's age, sex, and education as covariates). The *p* value for statistical significance was set at <0.05. All statistical analyses of demographic data and DTI indices were performed using SPSS software, version 17, for Windows (SPSS, Chicago, IL, USA).

#### 2.6.2. Group Comparison of FA Values between Healthy Controls and Patients with PD

The SPM8 (Statistical Parametric Mapping; http://www.fil.ion.ucl.ac.uk/spm/; University College London, London, UK) software package in Matlab R2010a (Mathworks, Natick, MA, USA) was used for voxelwise group comparisons. The FA images after normalization and smoothing were analyzed using SPM8 within the framework of a General Linear Model. ANCOVA with age and sex as covariates was performed to check the FA differences between the groups. The FA threshold of the mean WM was set at 0.2 to exclude “contaminated voxels” with composition of gray matter or cerebrospinal fluid. The statistical threshold was set at an uncorrected *p* < 0.005, with a cluster of >200 contiguous voxels. The extended cluster size was arbitrary and was used to putatively detect significant differences between groups with a cluster size <200 voxels, which might not represent reliable findings [25]. Additionally, multiple comparisons of FA were corrected by AlphaSim (FWE) to control for type I errors [22–24, 26] with the threshold at *p* < 0.05 and cluster size >1871.

The FSL atlas tool (https://www.fmrib.ox.ac.uk/fsl/fslwiki/Atlases) was facilitated for determining the most probable fiber tracts and anatomic location of each significant cluster.

#### 2.6.3. Analysis of Region of Interest

Region of interest (ROI) analyses were used to access the mean FA value of each significantly different area between the groups based on whole-brain voxelwise comparisons. The ROI masks were extracted by Marsbar toolbox (http://marsbar.sourceforge.net/download.html) [5, 27, 28]. All mean DTI-related indices of these areas were compared between groups by multivariate analysis of covariance, with age, sex, and education as covariates. Significance was set at a Bonferroni correction *p* < 0.05.

#### 2.6.4. Correlations among Demographic Data, Cardiovascular Autonomic Parameters, DTI Indices, and Plasma DNA

Partial correlation analysis was conducted to check the relationships among demographic data, cardiovascular autonomic parameters, and plasma DNA levels of PD groups after controlling for age and sex. To minimize the influence of education level on white matter integrity, partial correlations of DTI indices-related items were demonstrated after controlling age, sex, and education level.

#### 2.6.5. Mediation Analysis

A single-level three-variable mediation model was used to investigate the possible relationships among cardiac autonomic dysfunction, serum oxidative stress, and microstructural white matter (WM) changes by using the PROCESS macro with an accelerated bias-corrected bootstrap test of statistical significance used (5000 bootstrap samples) for SPSS [29]. Mediation analysis tests whether the direct effect of an independent variable on a dependent variable can be explained by the indirect influence of the mediating variable. A significant mediator is one whose inclusion as an intermediate variable significantly affects the relationship between the independent and dependent variables. The statistical significance threshold in Sobel test was set at 0.05 for all the relevant paths [30].

## 3. Results

### 3.1. The Demographic Data of Participants and the Disease Severity of PD Patients

The demographic data of the participants are shown in Table 1. The patient and normal control groups had similar mean age and sex distribution (age: *p* = 0.521; sex: *p* = 0.775). The distributions of the scales in UPDRS I, UPDRS II, UPDRS III, UPDRS 176, H&Y, and S&E are all demonstrated.

### 3.2. Between-Group Differences in Cardiovascular Autonomic Parameters

The cardiovascular autonomic parameters of the participants are listed in Table 1. Compared to the normal controls, the PD patients had significantly reduced BRS (mean ± standard deviation; 6.24 ± 3.18 versus 4.40 ± 2.62; *p* = 0.028).

### 3.3. Between-Group Difference in Regional WM Integrity Aberrances

**Figure 1:**
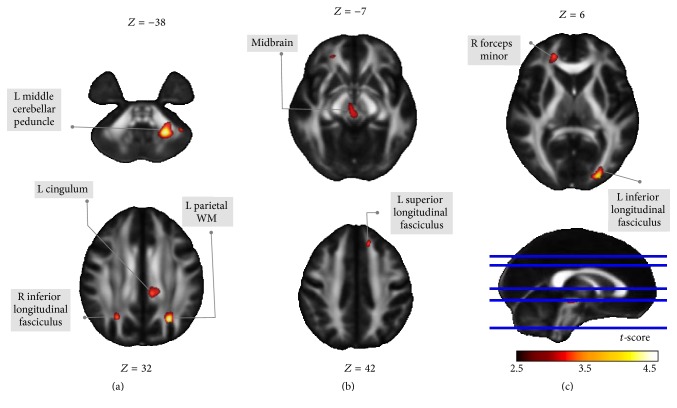
Patients with PD had lower FA values in several anatomic locations, including the left middle cerebellar peduncle, midbrain, forceps minor of the right frontal lobe, inferior longitudinal fasciculus of the left occipital lobe, left cingulum, left parietal WM, inferior longitudinal fasciculus of the right parietal lobe, and superior longitudinal fasciculus of the left frontal lobe. Among these anatomical locations with lower FA, the statistical results of the left parietal WM, left middle cerebellar peduncle, and left cingulum could pass the multiple comparisons corrected by AlphaSim at *p* < 0.05 and cluster size >1871.

The locations and spatial extents of anatomical regions with significant differences in FA map between the two groups are presented in Table 2 and Figure 1. Although these DTI indices are not independent, there are still different meanings for each of them which are discussed separately [31]. Patients with PD had lower FA values in the left parietal WM, left middle cerebellar peduncle, inferior longitudinal fasciculus of the left occipital and right parietal lobe, left cingulum, forceps minor of the right frontal lobe, superior longitudinal fasciculus of the left frontal lobe, and midbrain.

These lower FA values in PD were also associated with changes in other diffusivity indices, including the following: (1) increased MD and RD values in the left parietal WM, left cingulum, and inferior longitudinal fasciculus of the left occipital and right parietal lobe; (2) increased RD values in the forceps minor of the right frontal lobe and superior longitudinal fasciculus of the left frontal lobe; (3) reduced AD values in the left middle cerebellar peduncle; (4) increased MD, AD, and RD values in the midbrain.

### 3.4. Between-Group Differences in Plasma Nuclear and Mitochondrial DNA

**Figure 2:**
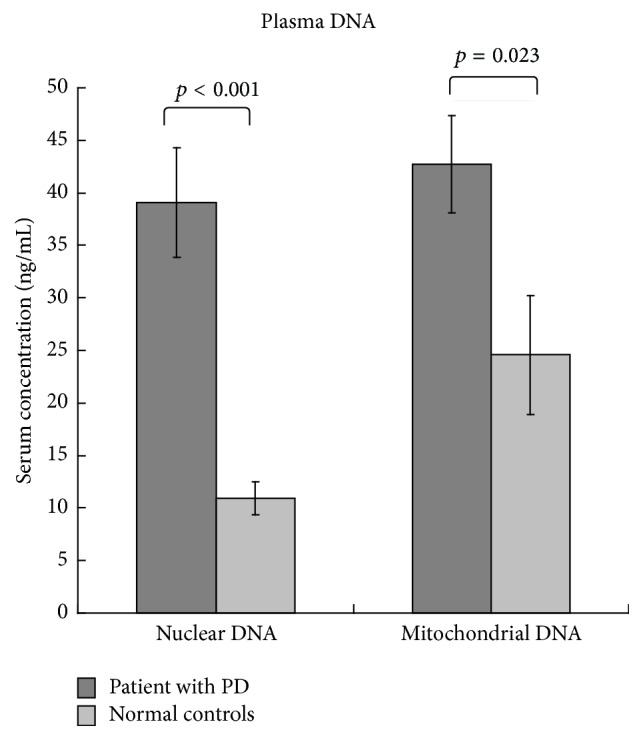
Compared to the controls, PD patients exhibited higher nuclear DNA level and mitochondrial DNA level.

The plasma nuclear and mitochondrial DNA levels of the participants are listed in Table 1 and Figure 2. PD patients (compared with controls) exhibited higher nuclear DNA level (mean ± standard deviation; 41.31 ± 29.95 versus 10.92 ± 8.17; *p* < 0.001) and mitochondrial DNA level (mean ± standard deviation; 42.70 ± 26.01 versus 24.56 ± 28.80; *p* = 0.023).

### 3.5. Correlations of Cardiovascular Autonomic Parameters, WM Integrity, and Plasma DNA Level

The summary of correlations with their *R*/*p* values in this study was listed in Table 3.

#### 3.5.1. Correlations Related to WM Integrity (FA, MD, AD, and RD)

*Correlations of FA Values with Cardiovascular Autonomic Parameters and Plasma DNA Level*. The FA values in the left middle cerebellar peduncle were negatively correlated with nuclear and mitochondrial DNA levels.

*Correlations of MD Values with Cardiovascular Autonomic Parameters and Plasma DNA Level*. The MD values in the left cingulum were positively correlated with baroreflex sensitivity and negatively correlated with serum nuclear DNA simultaneously.

*Correlations of AD Values with Cardiovascular Autonomic Parameters and Plasma DNA Level*. The AD values in the left cingulum were positively correlated with the serum nuclear DNA level. The AD values in superior longitudinal fasciculus of left frontal lobe were positively correlated with the serum mitochondrial DNA level.

*Correlations of RD Values with Cardiovascular Autonomic Parameters and Plasma DNA Level*. The RD values in the left cingulum were simultaneously positively correlated with baroreflex sensitivity and negatively correlated with serum nuclear DNA.

### 3.6. Correlation between Cardiovascular Autonomic Parameters and Plasma DNA Level

The reduced BRS was associated with increased serum nuclear DNA level (*r* = −0.535, *p* = 0.004). However, there is no significant correlation between BRS and mitochondrial DNA levels.

### 3.7. Mediation Analysis for Cardiovascular Autonomic Parameters, WM Integrity, and Serum Nuclear DNA Level

The primary hypothesis of this analysis asks whether the effect of the baroreflex sensitivity (independent variable) on the white matter integrity (dependent variable) was explained indirectly by the plasma nuclear DNA (mediator) with a significant group main effect. The path model jointly tested three effects of interest that are required if plasma nuclear DNA level links baroreflex sensitivity with the white matter integrity: (a) the effect of the independent variable (baroreflex sensitivity) on the mediator (plasma nuclear DNA) (indirect effect, path a); (b) the effect of the mediator on the dependent variable (DTI indices) by controlling the effect for the baroreflex sensitivity (indirect effect, path b); and (c) the mediation effect a*∗*b which is defined as the reduction of the relationship between the independent and dependent variables (baroreflex sensitivity-white matter integrity) (total relationship, path c) by including the mediator into the model (direct path, path c′).

**Figure 3:**
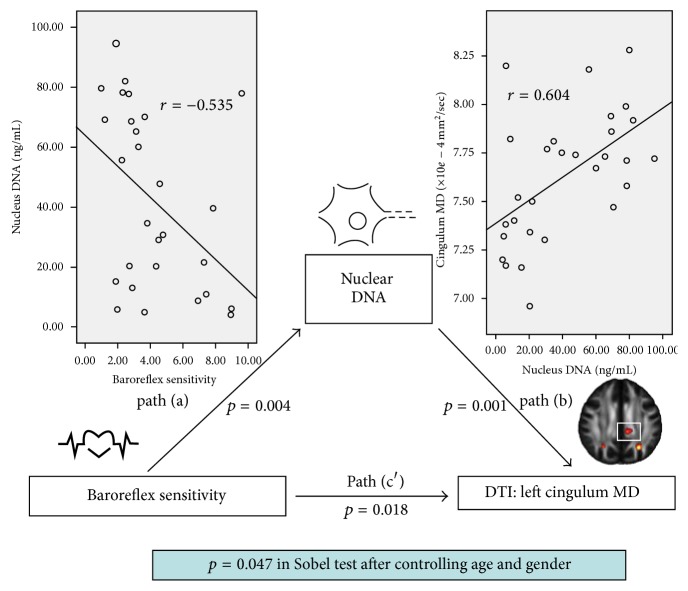
The mediation model in this study. Path (a) was defined as the effect of the independent variable (baroreflex sensitivity) on the mediator (nuclear DNA). Path (b) was the effect of the mediator (nuclear DNA) on the dependent variable (left cingulum MD) by controlling the effect of the baroreflex sensitivity. Path (c′) shows the direct path from independent variables (baroreflex sensitivity) to dependent variables (left cingulum MD). The mediation relationship passed the Sobel test after controlling age and sex.

For simplicity, we report a full list of the results from the present study that fulfill the three criteria cited previously. Consequently, the mediation statistics are performed only for the MD and RD indices of the left cingulum, BRS, and nuclear DNA. The mediation relationship was significant (*p* = 0.047 in Sobel test) after controlling age and sex (Figure 3).

## 4. Discussion

### 4.1. Summary

Consistent with previous studies and our hypothesis, PD patients have poor cardiovascular autonomic function, higher serum plasma DNA, and poor DTI indices in this study. Furthermore, the correlations among these clinical presentations are also demonstrated in this study. Finally, a mediation model among baroreflex sensitivity (independent variable), serum nuclear DNA (mediator), and cingulum MD (dependent variable) was discovered. This result suggests that the poor cardiovascular autonomic status in PD patients not only affects the WM microstructure directly but also increases the higher serum nuclear DNA level to influence the WM microstructure simultaneously.

### 4.2. The Origin of Poor Cardiovascular Status and Its Influence

The autonomic dysfunction in PD was previously demonstrated by reduced BRS [32] and linked to the possible central autonomic network in previous studies [3]. On the other hand, the poor BRS could cause hypoperfusion in the brain [33] and make sequential presentations, including WM integrity changes [34]. Our results also demonstrated that the poor BRS correlated with brain white matter microstructural deficits in PD patients. Cardiovascular autonomic function impairment in PD patient not only is a disease presentation but also turns out to be the origin of another vicious circle.

The vulnerable sites of hypoperfusion under poor BRS status included not only brain tissue [33] but also the peripheral tissue [35] with resultant cell death or apoptosis [36]. Plasma DNA is one of the most useful biomarkers of cell death or apoptosis [37]. Like PD [38], the higher plasma DNA level or higher oxidative stress in peripheral blood could be detected in many diseases with cell death or apoptosis [39, 40]. Owing to the great influence of hypoperfusion under poor BRS status, whether the BRS could be a therapeutic target or a biomarker for clinical outcome evaluation was examined [4].

### 4.3. Correlations between Oxidative Stress and DTI Indices

Significant oxidative stress was reported in several diseases related to cell damage or apoptosis, and the oxidative stress could be associated with the changes in brain microstructure [39]. Furthermore, the variant hypoxic pattern is representative of the interaction between oxidative stress and brain microstructural damage [5]. This concept was proven in the experimental animal model that showed oxidative stress could interfere with white matter renewal after prolonged cerebral hypoperfusion [41]. The higher oxidative stress level could be found in PD patients [8] and further worsen the brain DTI presentation by causing microstructural damage [38]. Consistent with previous studies, many of the correlations between the higher plasma DNA levels and poor DTI indices are in accordance with the hypothesis in patients with Parkinson's disease in this study.

### 4.4. The Possible Pathophysiology of the Mediation Model

Many correlations between the poor BRS presentation, elevated plasma DNA level, and poor DTI indices are demonstrated in this study. Different to the correlation relationship, a mediation model establishment provides a statistic way to expound the causal relationship with verifications and applications [29]. According to our results, a possible pathophysiology might be concluded. At first, the poor BRS status in PD patients caused a hypoperfusion environment that harms the cells, increases oxidative stress, and damages the microstructure of the brain. Simultaneously, the increased plasma DNA, in the form of oxidative stress, worsens brain microstructural damage further. Finally, as a result of passing the Sobel test in mediation, it has been suggested that the poor BRS status may not only increase the oxidative stress and change the brain DTI presentation in this same time, but also exacerbate the brain WM microstructural damage further, via elevated oxidative stress.

According to the criteria of mediation statistics, there are only two candidate data groups that could be enrolled: (1) MD of cingulum, BRS, and nuclear DN; (2) RD of cingulum, BRS, and nuclear DNA. The increased MD values reflect increased water diffusion in all directions, a result that suggests gliosis [31]. On the other hand, the increased RD values suggest increased water diffusion in the perpendicular direction, such as what would be seen in focal myelin disruption, loss, or alteration [42]. These two microstructural damage patterns were consistent with those reported in previous studies [43, 44].

According to previous reports, the cingulum is a vulnerable region of the brain in neurodegenerative diseases [45, 46]. It has thus been proposed that assessment of the cingulum fibers using diffusion tensor imaging could improve early diagnosis of neurodegenerative diseases [47]. It has also been proven, using an animal model, that the cingulum is highly susceptible to hypometabolic status [48]. As such, the results in this study indicating that the cingulum could be representative of such a possible pathophysiologic model are reasonable.

### 4.5. Limitations

Although this study presents a considerable body of evidence, the interpretation of these results must still be applied carefully. First, BRS is one of the causes of hypoperfusion and there are still other causes which influence intracranial perfusion status. Further arterial spin labeling could be conducted to provide more direct evidence for the possible pathophysiology. Second, plasma DNA was used to represent the oxidative stress status of these PD patients, but there is no enough evidence to suggest it is the only factor that affects the brain WM. It is possible that there are one or more oxidative biomarkers which could influence the pathophysiology more directly. Third, the details of the pathophysiology are far more complicated than we imagine. A relatively small sample size with temporal relationships or as the disease progresses could influence the results. This result provides us with a possible pathophysiology but is not the only path of PD pathogenesis. Last, unlike a de novo or longitudinal study, confounding factors such as medication, physical status, or comorbidity that may influence the oxidative stress cannot be totally ruled out in the observational study.

## 5. Conclusions

The coexistence of poor cardiovascular function, higher oxidative stress status, and extensive structural deficits is demonstrated in PD patients. It is been highlighted that these presentations are closely related to each other in this study. The mediation results provided the possible pathophysiology for how the poor baroreflex sensitivity and higher oxidative stress adversely impacted the WM integrity. This model could provide us with a piece of puzzle for the entire PD pathogenesis.

## Figures and Tables

**Table 1:** Demographic characteristics of PD patients and control subjects.

	PD	Normal control	*p*
Number	29	26	
Sex (M : F)	20 : 9	19 : 7	0.775
Age	61.51 ± 8.27	60.11 ± 7.77	0.521
UPDRS I	3.96 ± 2.97		
UPDRS II	12.21 ± 7.26		
UPDRS III	27.03 ± 13.20		
UPDRS 176	43.10 ± 21.93		
Modified Hoehn and Yahr staging	2.36 ± 0.97		
Schwab and England (S&E) scale	81.03 ± 13.98		

*Oxidative stress biomarkers*			
Nuclear DNA (ng/mL)	41.31 ± 29.95	10.92 ± 8.17	<0.001^*∗*^
Mitochondrial DNA (ng/mL)	42.70 ± 26.01	24.56 ± 28.80	0.023^*∗*^

*Autonomic parameters*			
HR_DB	7.38 ± 8.35	8.96 ± 6.00	0.480
BRS	4.40 ± 2.62	6.24 ± 3.18	0.028^*∗*^
LF/HF ratio	2.15 ± 2.00	1.77 ± 1.52	0.549
LF	54.94 ± 24.76	52.60 ± 22.91	0.815
HF	45.05 ± 24.76	47.33 ± 22.98	0.821

Sex data were compared by Pearson chi-square test.

Age data were compared by independent *t*-test.

Oxidative stress biomarkers and autonomic parameters were compared by analysis of covariance (ANCOVA) after controlling for age and sex. Data are presented as mean ± standard deviation.

^*∗*^ *p* < 0.05.

PD, Parkinson's disease; HR_DB, heart rate response to deep breathing; BRS, baroreflex sensitivity; LF, low frequency; HF, high frequency.

**Table 2:** Regions showing fractional anisotropy differences between patients with Parkinson's disease (PD) and control subjects (NC).

MNI atlas coordinates			Voxel size	White matter tract	FA mean (SD), *p* < 0.001		*t*_max_	Diffusivity values (PD-NC) (×10^−6^)		
*X*	*Y*	*Z*			Controls	PD		MD (*p* = 0.004)	AD (*p* = 0.016)	RD (*p* = 0.002)
				*Decreased FA in PD patients *versus* Controls*						
*−26*	*−63*	*33*	*1914*	*Parietal WM, left*	*0.449 (0.031)*	*0.406 (0.034)*	*4.46*	*27* ^*∗*^	*−9*	*44* ^*∗*^
*−22*	*−53*	*−38*	*2698*	*Middle cerebellar peduncle, left*	*0.427 (0.023)*	*0.339 (0.026)*	*4.41*	*2*	*−27* ^*∗*^	*17*
−24	−87	6	647	Inferior longitudinal fasciculus, left occipital lobe	0.375 (0.027)	0.338 (0.023)	4.05	30^*∗*^	−12	51^*∗*^
27	−61	32	522	Inferior longitudinal fasciculus, right parietal lobe	0.425 (0.033)	0.381 (0.045)	3.49	29^*∗*^	−14	50^*∗*^
*−9*	*−35*	*32*	*2350*	*Cingulum, left*	*0.642 (0.030)*	*0.612 (0.025)*	*3.45*	*35* ^*∗*^	*21*	*42* ^*∗*^
20	42	4	902	Forceps minor, right frontal lobe	0.474 (0.033)	0.432 (0.048)	3.44	17	−34	43^*∗*^
−12	30	42	203	Superior longitudinal fasciculus, left frontal lobe	0.349 (0.030)	0.319 (0.029)	3.38	25	3	35^*∗*^
3	−20	−7	563	Midbrain	0.364 (0.034)	0.327 (0.043)	3.21	230^*∗*^	236^*∗*^	228^*∗*^

Location of maximum effect (uncorrected *p* < 0.005, cluster size >200) was shown in the Montreal Neurological Institute (MNI) space.

Group FA mean values in each cluster are presented as mean (standard deviation).

Passing the AlphaSim correction (*p* < 0.05, cluster size >1871) is identified by italic.

The FA, MD, AD, and RD values in the regions of interest were further compared between two groups by analysis of covariance after controlling for age, sex, and education.

^*∗*^ *p* < 0.05 with a Bonferroni correction, accounting for multiple ROI comparisons.

WM, white matter; FA, fractional anisotropy; AD, axial diffusivity; MD, mean diffusivity; RD, radial diffusivity.

**Table 3:** Correlations among diffusion tensor abnormalities, oxidative stress, and cardiovascular autonomic parameters.

WM tract	Correlation of clinical variable (*r*/*p value*)		
	Baroreflex sensitivity (BRS)	Nuclear DNA	Mitochondrial DNA
*Left parietal WM *			
FA	0.280/0.158	−0.286/0.148	−0.296/0.134
MD	−0.272/0.410	0.165/0.410	0.096/0.633
AD	−0.104/0.604	−0.026/0.896	−0.097/0.629
RD	−0.315/0.109	0.241/0.226	0.188/0.347
*Left middle cerebellar peduncle *			
FA	0.370/0.058	−0.514/0.006^**∗∗**^	−0.546/0.003^**∗∗**^
MD	−0.202/0.312	0.125/0.534	0.248/0.211
AD	−0.007/0.971	−0.118/0.557	0.034/0.865
RD	−0.311/0.114	0.280/0.157	0.369/0.058
*Left cingulum *			
FA	0.199/0.319	−0.305/0.122	0.054/0.789
MD	−0.452/0.018^**∗**^	0.604/0.001^**∗∗**^	0.207/0.299
AD	−0.377/0.052	0.562/0.002^**∗∗**^	0.314/0.111
RD	−0.415/0.031^**∗**^	0.519/0.006^**∗∗**^	0.110/0.583

Correlations among diffusion tensor abnormalities, oxidative stress biomarkers, and cardiovascular autonomic parameters were performed by partial correlation after controlling for age and sex.

^*∗*^ *p* < 0.05, uncorrected.

^*∗∗*^ *p* < 0.05 with a Bonferroni correction, accounting for multiple region of interest comparisons.

FA, fractional anisotropy; AD, axial diffusivity; RD, radial diffusivity; BRS, baroreflex sensitivity; WM, white matter.
